# Hereditary orotic aciduria (HOA): A novel uridine-5-monophosphate synthase (*UMPS*) mutation

**DOI:** 10.1016/j.ymgmr.2020.100703

**Published:** 2021-01-09

**Authors:** Hebah S. Al Absi, Stephanie Sacharow, Naser Al Zein, Aisha Al Shamsi, Amal Al Teneiji

**Affiliations:** a Department of Pediatrics, Sheikh Khalifa Medical City, Abu Dhabi, United Arab Emirates; b Division of Genetics and Genomics, Boston Children's Hospital, Harvard Medical School, Boston, MA, USA; c Department of Pediatrics, Division of Hematology and Oncology, Sheikh Khalifa Medical City, Abu Dhabi, United Arab Emirates; d Department of Pediatrics, Division of Genetics and Metabolic, Tawam Hospital, Al Ain, United Arab Emirates; e Department of Pediatrics, Division of Metabolic Genetics, Sheikh Khalifa Medical City, Abu Dhabi, United Arab Emirates

**Keywords:** Orotic aciduria, Uridine-5-monophosphate synthase, Immunodeficiency

## Abstract

Hereditary orotic aciduria (HOA) is a very rare inborn error of pyrimidine metabolism. It results from a defect of the uridine-5-monophosphate synthase (*UMPS*) gene. To date, only about twenty patients have been described. We report a case of HOA with a novel variant in the *UMPS* gene. A 17-year-old Emirati girl was born to first-cousin parents. During the first year, she had recurrent, severe infections including disseminated varicella. After evaluation for immunodeficiency, an impression of immunodeficiency of unknown etiology was presumed. Frequent episodes of pancytopenia were also noted. Bone marrow biopsy showed trilineage megaloblastoid maturation with dysplastic changes that were refractory to hematinic therapy. Also, she was noted to have failure to thrive, developmental delay and epilepsy. She was referred to the Genetics clinic where whole-exome sequencing (WES) was done and showed a novel homozygous variant in the *UMPS* gene confirming a diagnosis of HOA. She was started on uridine triacetate after which she showed clinical, hematologic and biochemical improvement. Although extremely rare, hereditary orotic aciduria should be suspected in any child with megaloblastic bone marrow, immunodeficiency or when developmental delay and anemia coexist.

## 1 Introduction

Hereditary orotic aciduria (HOA) (OMIM #258900) is a rare inborn error of pyrimidine metabolism with autosomal recessive inheritance [2]. It is the only known enzyme deficiency of the pyrimidine biosynthetic pathway, resulting from a deficiency in one or both of the activities of the bifunctional enzyme uridine-5-monophosphate synthase (UMPS) (EC 4.1.1.23) encoded by the *UMPS* gene. In the first reaction, orotate phosphoribosyltransferase (OPRTase) converts orotate to orotidine monophosphate via ribosylation. In the second step, orotidine decarboxylase (OMPdecase) decarboxylates orotidine monophosphate to uridine monophosphate [1]. The presumed cause of HOA is biallelic missense mutations resulting in decreased levels of the enzyme and impaired substrate binding [7]. *UMPS* defects lead to the accumulation of orotate (OA) and/or of orotidine monophosphate (OMP), which will eventually be excreted in the urine [8]. The hallmarks of the disease are a megaloblastic bone marrow that is refractory to hematinic therapy, accompanied by a markedly increased excretion of orotic acid in the urine. Immunodeficiency, developmental delay and failure to thrive have been observed [2,9]. To date, about twenty patients with HOA have been reported. Of these, only one case described an associated epileptic disorder [4].

## 2 Clinical history

We report a case of a 17-year-old Emirati girl who was born at a local hospital in 2003. She was born extremely premature at 28 weeks of gestation and was admitted to the newborn intensive care unit (NICU), where she stayed for 100 days. During the first year of her life, she required many admissions secondary to repeated infections. Her most serious infection was disseminated varicella-zoster skin infection at 7 months of age. This infection was complicated by pseudomonas-associated ecthyma gangrenosum which evolved into secondary septicemia, disseminated intravascular coagulopathy, varicella-associated hepatitis, and acute liver failure.

### 2.1 Immunologic evaluation

Due to the severity of her infection, she was evaluated for immunodeficiency. Serum IgM and IgG levels were elevated for age and flow cytometric assessment of lymphocyte subsets suggested a combined deficiency of B and T cells. The high immunoglobulin levels and the low lymphocyte counts were most likely deviated by the septic process. Tests were repeated when healthy and results confirmed impaired cellular immunity with normal humoral immunity. Further evaluation for immunodeficiency yielded no specific diagnosis and the impression of an unidentified immunodeficiency /immune dysregulation syndrome was given. Since then, she had been started on a prophylactic dose of Trimethoprim/sulfamethoxazole (TMP/SMX).

### 2.2 Hematologic evaluation

During her follow-ups, it was noted that her complete blood count (CBC) was significant for intermittent episodes of pancytopenia manifesting as anemia, leukopenia and mild thrombocytopenia (Table 1). Anemia was moderate in severity and characterized by low mean corpuscular hemoglobin (MCV), low-normal reticulocyte count and a very high red blood cell distribution width (RDW). Peripheral blood smears mainly showed microcytic hypochromic red blood cells with pronounced anisopoikilocytosis. Iron studies showed high Ferritin with normal iron, transferrin, transferrin saturation, and total iron-binding capacity. Hemoglobin electrophoresis confirmed a diagnosis of beta-thalassemia trait. Although iron studies were normal, she was started on a trial of iron therapy (ferrous sulfate 50 mg BID) but with poor clinical and hematologic response. As a result of the persistent pancytopenia, it was a necessity to do bone marrow aspirate and biopsy. Bone marrow results are shown in Table 1. Standard cytogenetics for myelodysplastic syndrome (MDS) came back negative with no evidence of translocations, rearrangements, gains or lossses. The megaloblastic bone marrow urged measurement for levels of folic acid and vitamin B12, which were found to be high. Despite that, she was tried on supplements of folic acid (1 mg daily) and vitamin B12 but with no response. Then, she was tried on pyridoxine supplements of (100 mg daily) to cover for possible sideroblastic anemia but also without response. Dietary supplements were stopped eventually. Follow-up bone marrow aspirate and biopsy were done at 10 years of age and showed features similar to those found in the previous one (Table 1).The impression was dyserythropoietic anemia secondary to immunodeficiency.

**Table 1:** Peripheral blood counts and bone marrow aspiration.

Variable	Age		
	6 years	10 years	13 years (6 months post treatment)
Peripheral blood count			
Hemoglobin (g/dL)	10.9 (11.5–15.5) (L)	6.8 (11.5–15.5) (L)	12.3 (12.0–16.0) (N)
Reticulocytes RDW (%)	0.2 (0.5–1.0) (L) 40 (11.5–15.0) (H)	0.4 (0.5–1.0) (L) 42 (11.5–15.0) (H)	1.2 (0.5–1.0) (H) 18 (11.5–14.0) (H)
White blood cell count (×10^9/L)	1.28 (5–14.5) (L)	4.1 (4.5–13.5) (L)	6.0 (4.5–13) (N)
Platelet count (×10^9/L)	148 (150–350) (L)	118 (150–350) (L)	231 (150–350) (N)

Bone marrow results			
	• Significant trilineage megaloblastoid maturation and dysplastic changes • Nuclear irregularity and occasional binuclearity • Hypochromic, microcytic anemia with severe anisopoikilocytosis • Marked increase in iron stores with increased sideroblasts • No increase in blast percentage		• Mild panhypoplasia with mild megaloblastic changes. • No dysplastic changes

Data from B Nathan, D. G., Orkin, S. H., & Oski, F. A. (1998). Nathan and Oski's hematology of infancy and childhood. Philadelphia: W.B. Saunders.

### 2.3 Associated findings

Our patient's disease was also associated with failure to thrive and a weight persistently below the 3rd percentile, microcephaly with a head circumference at the 1st percentile (-2.3 SD), moderate intellectual disability and developmental delay. At the age of 9 years, she had a seizure after a near-drowning episode, and subsequently started having generalized tonic-clonic seizures and was diagnosed with epilepsy. Consequently, she was started on antiepileptic drugs including valproic acid and clonazepam. The electroencephalogram (EEG) was very abnormal and showed irregular generalized spike and wave complexes with shifting predominance. Subsequent brain MRI demonstrated abnormal gyral folding along the anterior and inferior frontal lobes bilaterally with possible polymicrogyria, global cerebellar volume loss, and abnormality in the left frontal lobe periventricular white matter.

### 2.4 Family history

There was parental consanguinity with the parents being first cousins. The parents and the five siblings were healthy. Family history from maternal and paternal sides was significant for multiple miscarriages and neurological diseases of unknown etiologies.

### 2.5 Further investigations

Later on, she went to the United States for a second opinion. She was referred to the Genetics clinic for evaluation, during which whole-exome sequencing (WES) was performed.

### 2.6 Genetic analysis

After obtaining informed consent, blood samples were collected and processed for genomic DNA isolation. WES was performed using a trio-based approach (patient, mother, and father). The analysis demonstrated a previously unreported homozygous pathogenic variant in the *UMPS* gene (c.1010C > G; A337G) confirming a diagnosis of HOA. Both parents were found to be carriers of the same *UMPS* gene variant. Parents were clinically asymptomatic. Further findings of crystalluria and a significant urinary excretion of orotic acid in our patient (>266.1 mmol/mol creatinine; reference range 0.2–1.5) supported the diagnosis.

### 2.7 Initial management and outcome

**Fig. 1:**
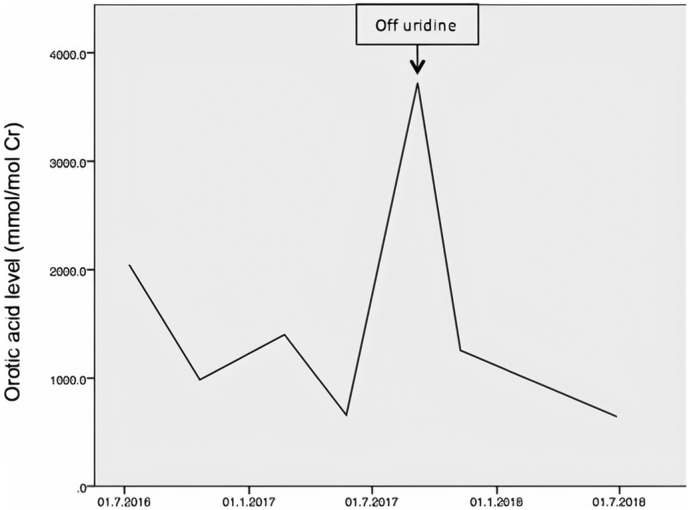
Urinary excretion rate of orotic acid after initiation of uridine therapy. Note the increased excretion that occurred when she was briefly off uridine.

She was started on uridine triacetate 2 g once daily (60 mg/kg body weight). The dose was increased to achieve the optimal therapeutic response, which was noted at 3.2 g (90 mg/kg body weight). She continued her management at our hospital with the metabolic team. Regular follow-ups showed a clinical, immunologic, hematologic and biochemical response. Her growth parameters, including weight and height, improved after starting her on uridine supplements. After 2 years of treatment, weight increased from below the 3^ed^ percentile to the 50th percentile. Height increased from the 10th percentile to the 25th percentile. CBC was done every 3 months along with a bone marrow biopsy when necessary (Table 1). Hematologic parameters showed normalization of hemoglobin levels with normal reticulocyte count and significant improvement in the RDW. Having a beta-thalassemia trait contributed to the persistently low MCV value. Platelet and WBC normalized. Lymphocyte count increased consistently during therapy and there was no reported increased susceptibility to infections. Bone marrow after 6 months of therapy showed significant improvement of the previously described megaloblastic changes without dysplasia. Urine levels of orotic acid were followed up every 3 months. During therapy, urinary excretion of orotic acid fell gradually (Fig. 1). The main issue we faced initially with our patient was the difficulty in obtaining uridine triacetate from abroad. She stayed off her medicine for 2 months, during this time she regressed. She developed fatigue and started to have breakthrough seizures in addition to an increase in urinary excretion of orotic acid (Fig. 1). Since restarting uridine triacetate, there were no breakthrough seizures reported. EEG was done and reported normal.

## 3 Discussion

Deficiencies in UMPS result in one of three clinical subtypes of HOA. Type I is caused by loss of both enzyme activities of the UMP enzyme. Type II is thought to be due to specific inactivation of OMPdecase. Type III, also called orotic aciduria without megaloblastic anemia (OAWA), is expected to be also secondary to the inactivation of OMPdecase [1]. Although orotic acid is an important precursor in the synthesis of pyrimidine, only minute amounts are found in normal blood and urine [6]. The urinary excretion of large amounts of orotic acid was first reported by Huguley et al. [5], occurring in a male child with refractory megaloblastic anemia. This child ultimately died from fatal varicella, whereas, notably, our patient had a life-threatening disseminated varicella infection. It has been reported that the main immunodeficiency in patients with HOA is the selective impairment in T cell function with intact humoral immunity. As a result, HOA was suggested for consideration under the spectrum of inborn errors of metabolism associated with immunodeficiency [3]. Nevertheless, our patient showed a dramatic increase in her lymphocyte count during therapy with a significant decrease in her susceptibility to infections. Neurological symptoms associated with HOA include; developmental delay and intellectual impairment [9]. The first patient with seizure-associated HOA was described by Grohmann et al. [4], proposing a link between UMPS deficiency and epilepsy. Here we describe another case with HOA and epilepsy supporting the suggested theory, though our patient's etiology is complicated by a concurrent history of extreme prematurity, a near-drowning episode, polymicrogyria, and parental consanguinity. However, being that uridine treatment caused resolution of seizures and lapse in treatment caused recurrence of seizures suggests that her epilepsy was indeed caused by HOA. The same factors that contributed to her seizures might have also caused her intellectual disability. Microcephaly and polymicrogyria are not reported in association with HOA and we think these can be related to very stormy clinical history and/or more likely another monogenic disease that has not been found yet.

Treatment is performed with the nucleoside uridine. The dosages described range from 50 to 200 mg/kg [9]. We monitored response to therapy in our patient by performing periodic examinations of peripheral blood films along with estimations of orotic acid excretion supplemented by bone marrow examinations when necessary. Our patient continued to demonstrate clinical response in the 2 years of uridine therapy, but some permanent intellectual disability persists. This emphasizes the importance of early diagnosis and treatment to ensure better cognitive development.

### 3.1 Conclusion

Although extremely rare, hereditary orotic aciduria should be suspected in any child with megaloblastic bone marrow, immunodeficiency or when developmental delay and anemia coexist. Early diagnosis and treatment are essential for normal development. In the case of our patient, establishing a diagnosis of HOA dramatically altered the disease course.

## Declaration of Competing Interest

The authors state no conflict of interest.
